# Quick Tanning

**Published:** 1875-04

**Authors:** 


QUICK TANNING.—A canny Scot has discovered that if a hide is immersed for four or five days in a mixture of vegetable or animal charcoal and water, of the consistency of a thin paste, the hair is entirely removed, and the leather made from a hide thus treated is of superior quality.

